# Supplementary material for: Trapped charge-driven degradation of perovskite solar cells
Source: Nat Commun. 2016 Nov 10;7:13422. doi: 10.1038/ncomms13422 (PMC5110646; doi:10.1038/ncomms13422)
Supplement: Supplementary Information — Supplementary Figures 1-16, Supplementary Table 1, Supplementary Notes 1-3 and Supplementary References. [file ncomms13422-s1.pdf]

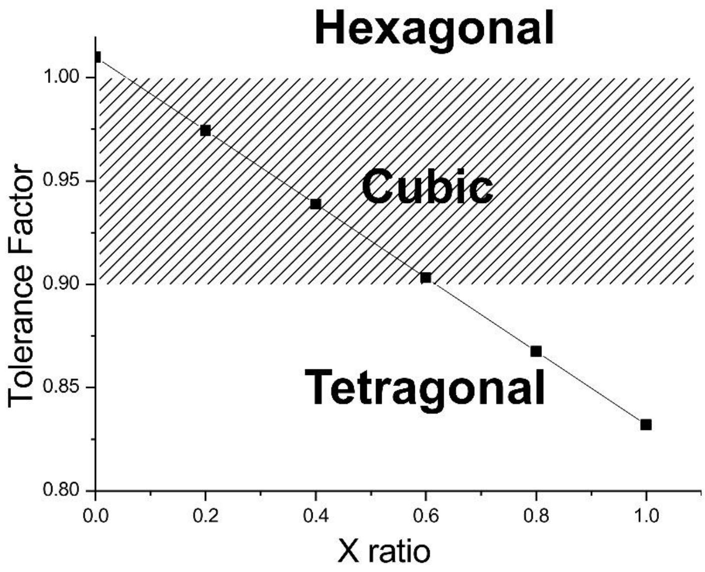

Supplementary Figure 1. Dependence of the Goldschmidt tolerance factor on the MA fraction (*x*) of $MA_xFA_{1-x}PbI_3$. Dashed zone means region for cubic structure (0.9 < t < 1).

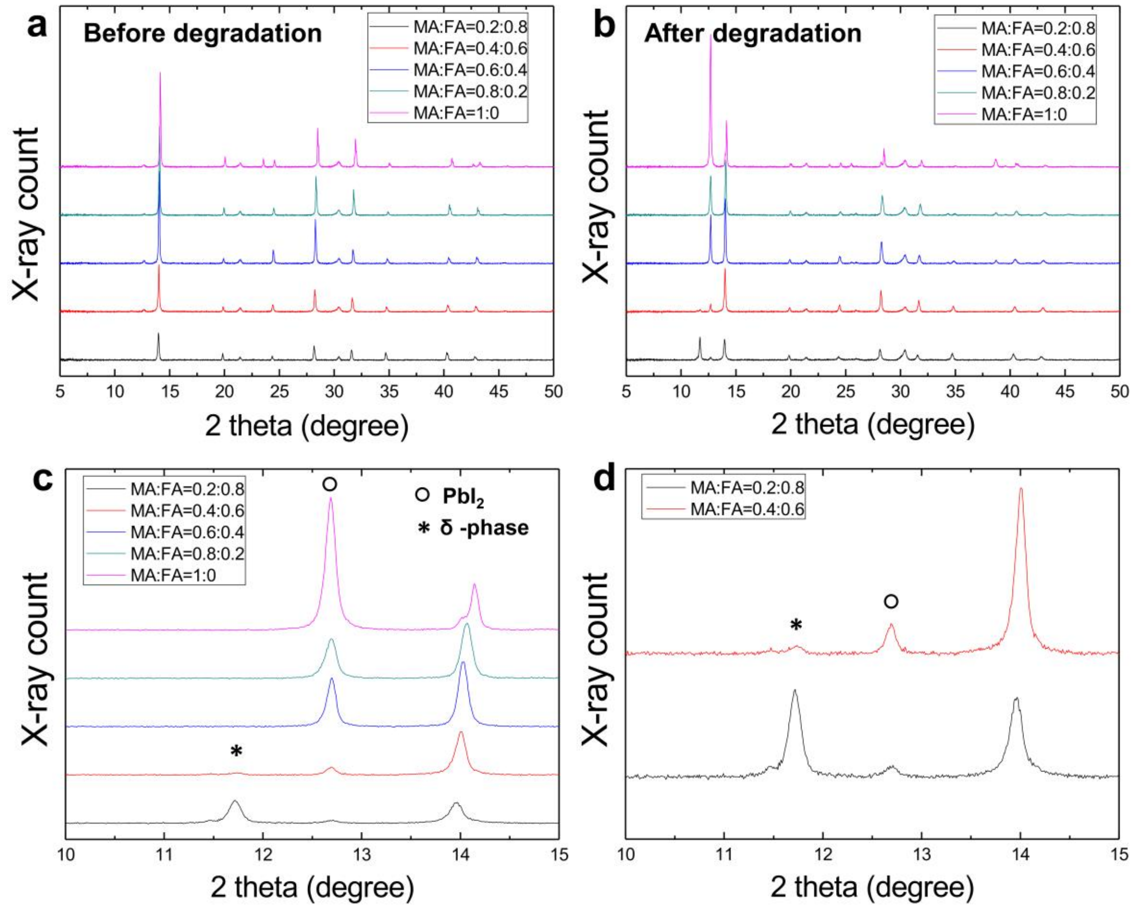

Supplementary Figure 2. X-ray diffraction patterns of five different mixed perovskite films coated on ITO glass (x=0.2 (black), 0.4 (red), 0.6 (blue), 0.8 (green), and 1 (pink)) (a) before and (b) after degradation under one sun illumination at 50 % relative humidity for 10 hours. (c) Magnified XRD patterns around the peaks originating from PbI_2 and non-perovskite δ–phase. (d) Magnified XRD patterns for x=0.2 and 0.4.

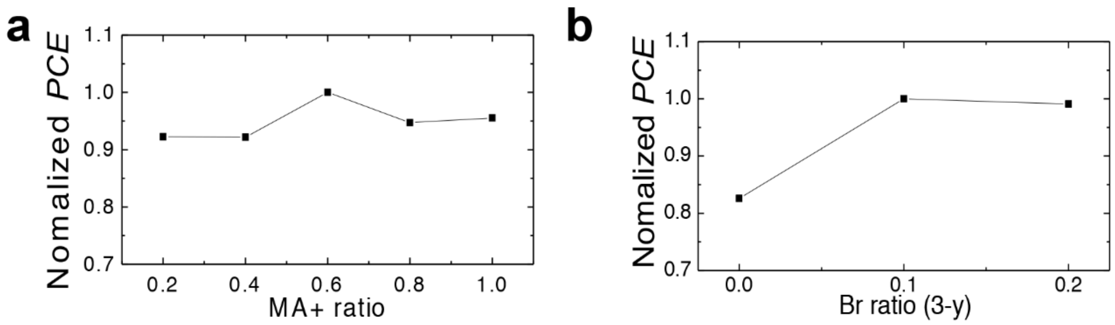

Supplementary Figure 3. Dependence of the normalized PCEs of ITO/C_{60}/Perovskite/Spiro-MeOTAD /A device on (a) the MA+ fraction (x), and (b) the Br- fraction (3-y)

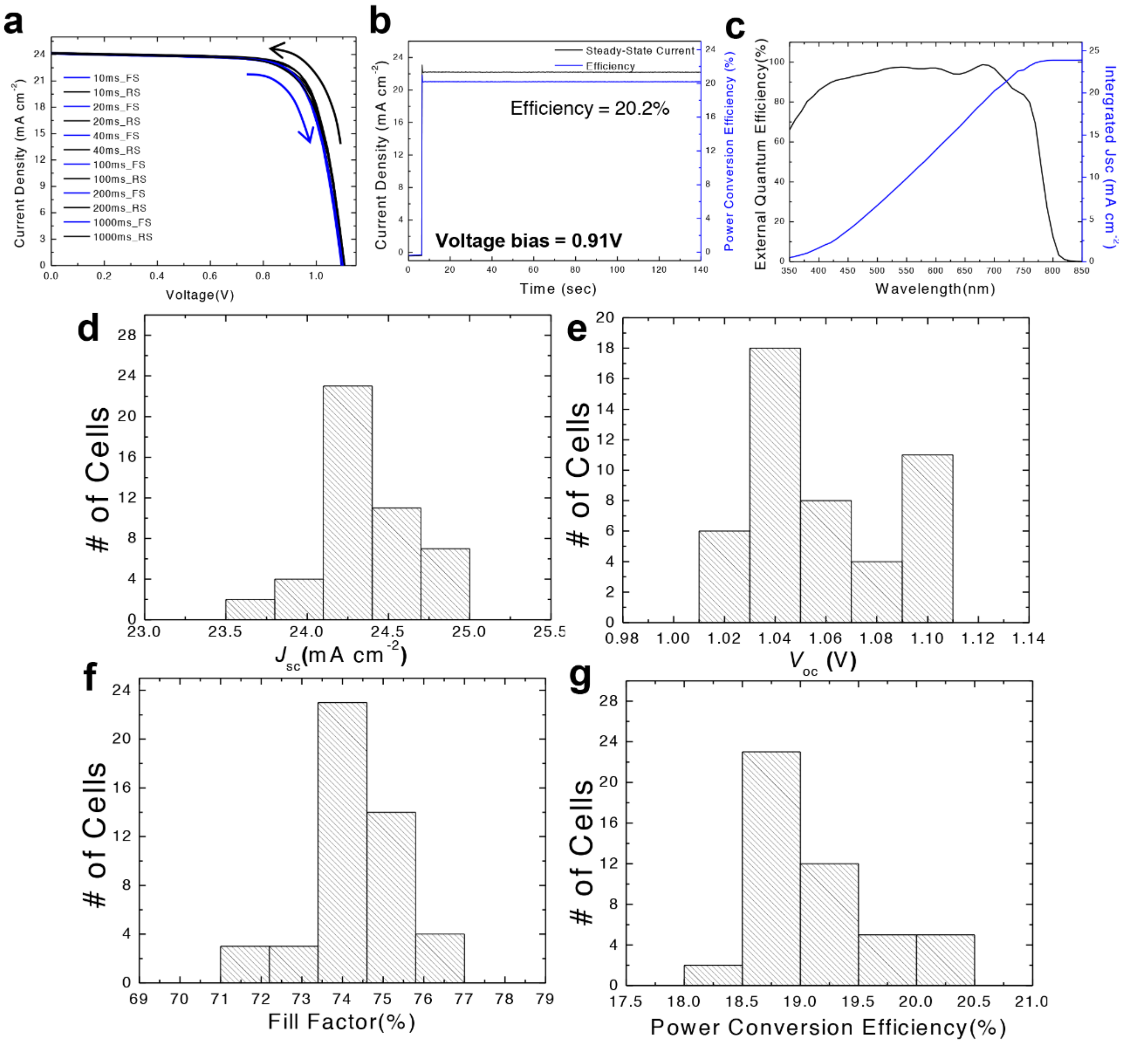

Supplementary Figure 4. Photovoltaic performance characteristics. (a) *J-V* curves of the best-performing device measured at various sweep delay times. (b) Stabilized photocurrent density (black) and power conversion efficiency (blue) measured at a bias voltage of 0.91V for 140 seconds. (c) External quantum efficiency (EQE) spectrum and the integrated J_{sc} estimated from the measured EQE. Histograms of (d) short-circuit current density (J_{sc}), (e) open-circuit voltage (V_{oc}), (f) fill factor (FF), and (g) power conversion efficiency (*PCE*) of 47 cells

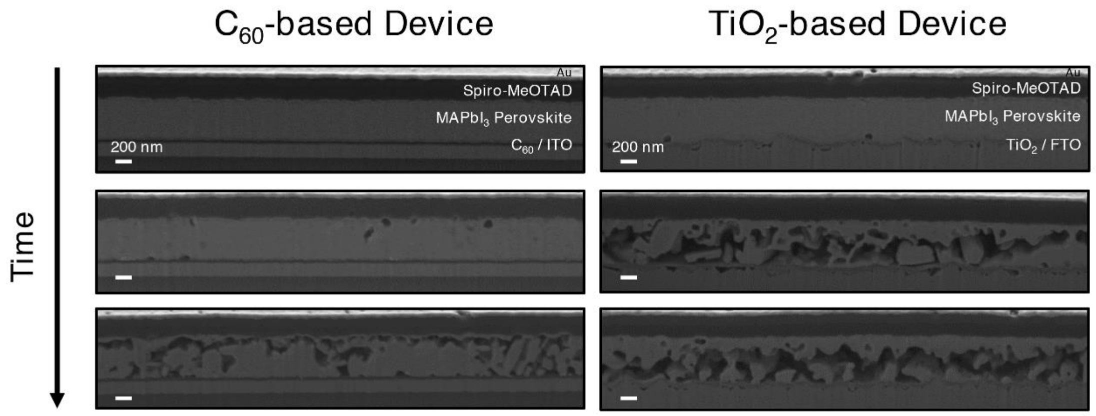

Supplementary Figure 5. Degradation patterns of the C_{60}- (left) TiO_2- (right) based devices employing $MAPbI_3$ perovskite, which were aged under one sun illumination in ambient conditions. Scale bars = 200 nm. As was shown in Fig. 1 for our mixed perovskite, the cell employing $MAPbI_3$ also showed the same pattern of degradation: for TiO_2-based cell, the degradation begins from the interface between $MAPbI_3$ and TiO_2 electron extraction layer, but for C_{60}-based cell, the degradation begins from the opposite side of interface between $MAPbI_3$ and Spiro-MeOTAD hole extraction layer.

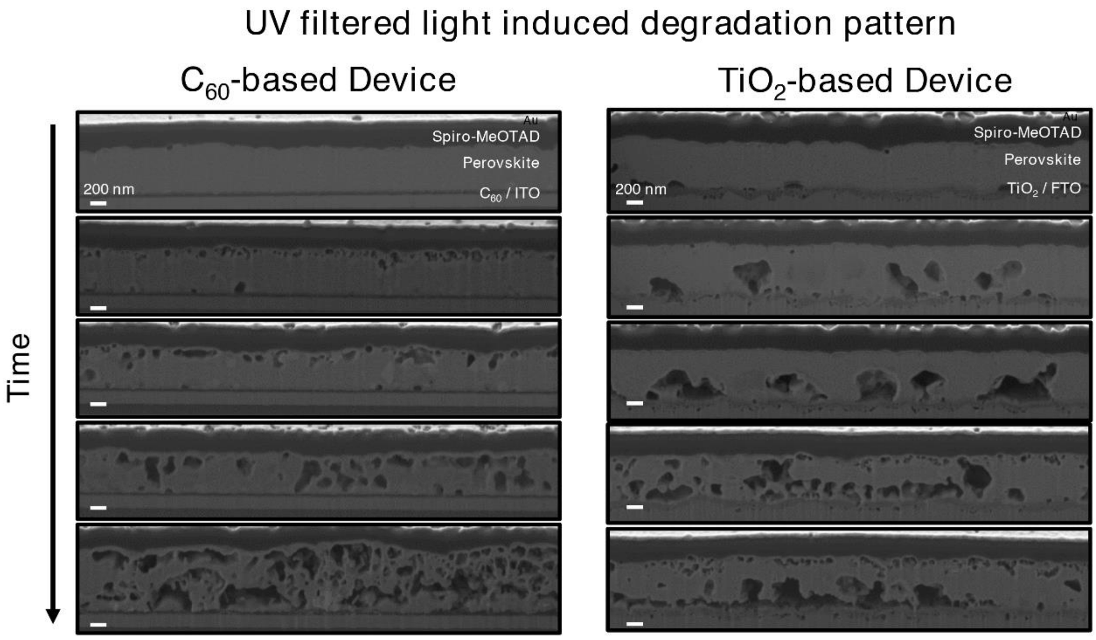

Supplementary Figure 6. Time evolution of the FIB-SEM cross-sectional images of the C_{60} (left) and TiO_2 (right) based devices employing $MA_{0.6}FA_{0.4}PbI_{2.9}Br_{0.1}$ perovskites, which were aged for 48 h under UV filtered (Edmund Optics, 425nm High Performance UV Longpass Filter) light illumination in ambient conditions. Scale bars = 200 nm.

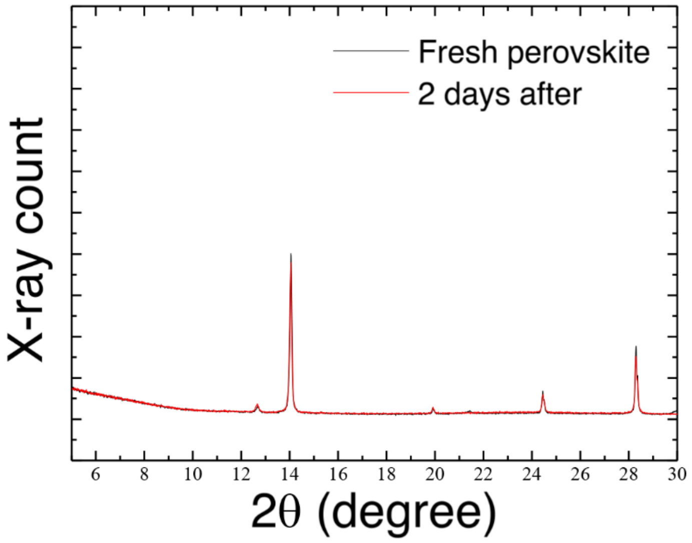

Supplementary Figure 7. XRD patterns of the perovskite film before (red) and after (black) aged at 90% relative humidity for 2 days.

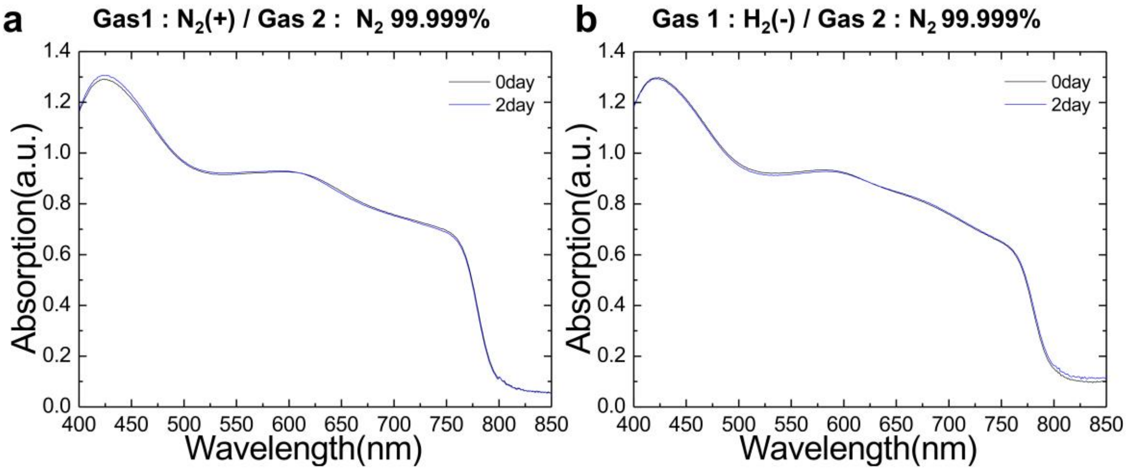

Supplementary Figure 8. Absorption spectra of the perovskite film under (**a**) continuous positive nitrogen and (**b**) negative hydrogen ion deposition in moisture-free dark condition.

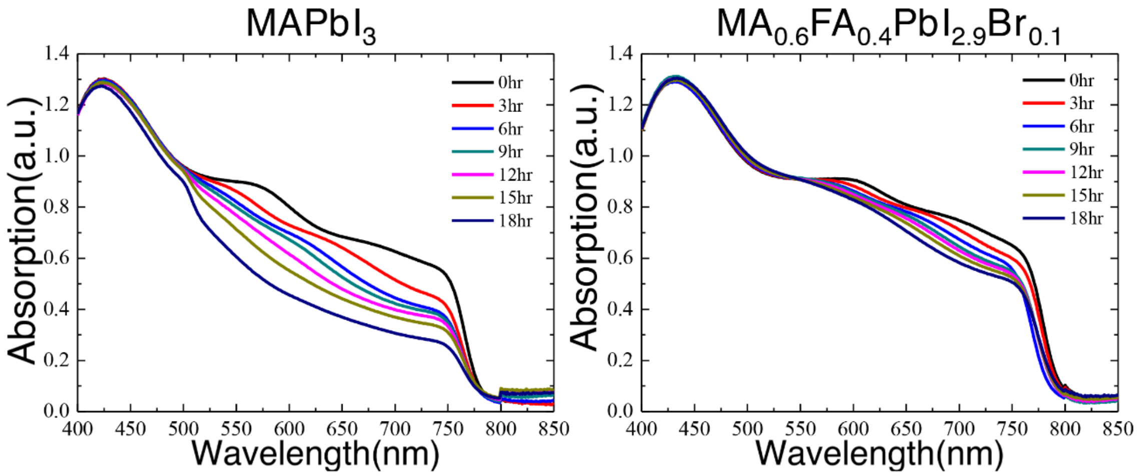

Supplementary Figure 9. Comparison of the degradation rates of $MAPbI_3$ and $MA_{0.6}FA_{0.4}PbI_{2.9}Br_{0.1}$ perovskite films at 40% relative humidity with positive nitrogen ion deposition. Absorption spectra were measured at an interval of 3 hr.

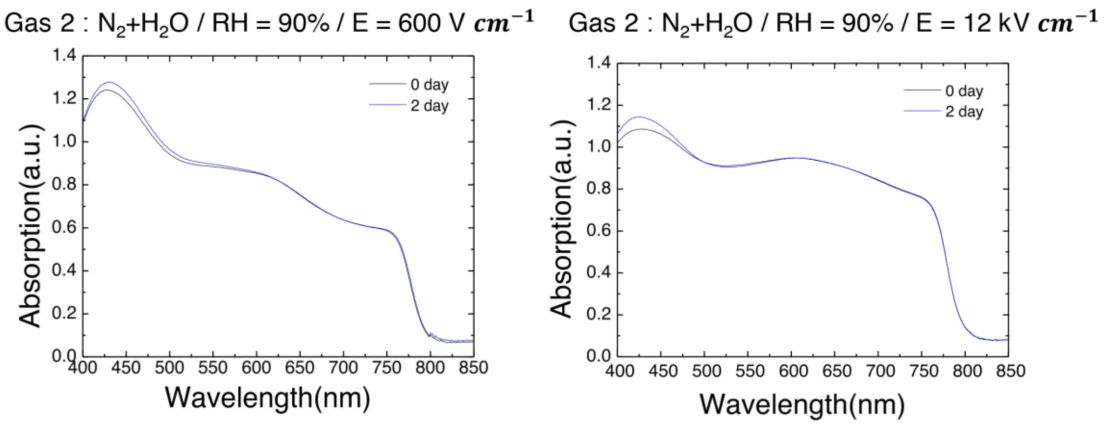

Supplementary Figure 10. The effect of non-contact high electric field on the degradation of the perovskite film at high relative humidity (90%) under dark condition.

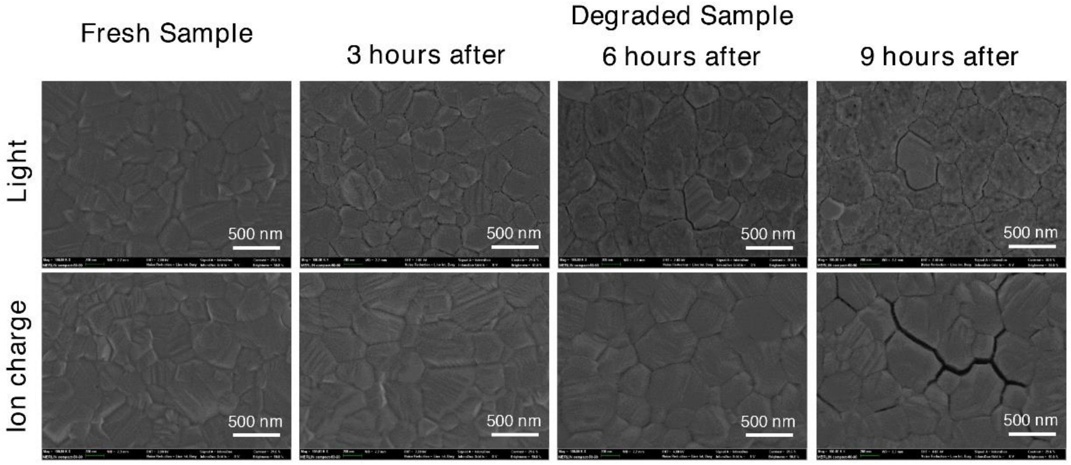

Supplementary Figure 11. The top-view SEM images of the fresh and degraded perovskite films. The degraded samples were aged for 9 hours under one sun light illumination (first row) and ion charge deposition (second row), respectively.

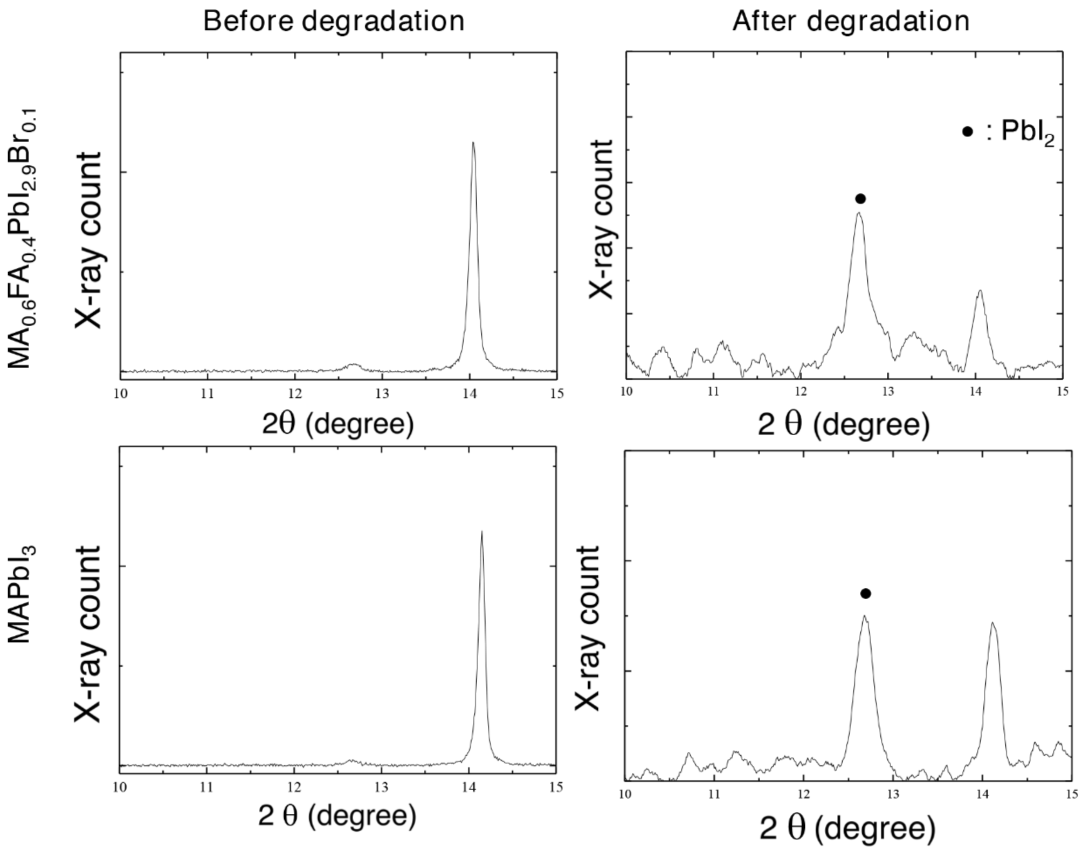

Supplementary Figure 12. XRD patterns of the $MA_{0.6}FA_{0.4}PbI_{2.9}Br_{0.1}$ and $MAPbI_3$ film before and after degradation by trapped charges in the presence of moisture.

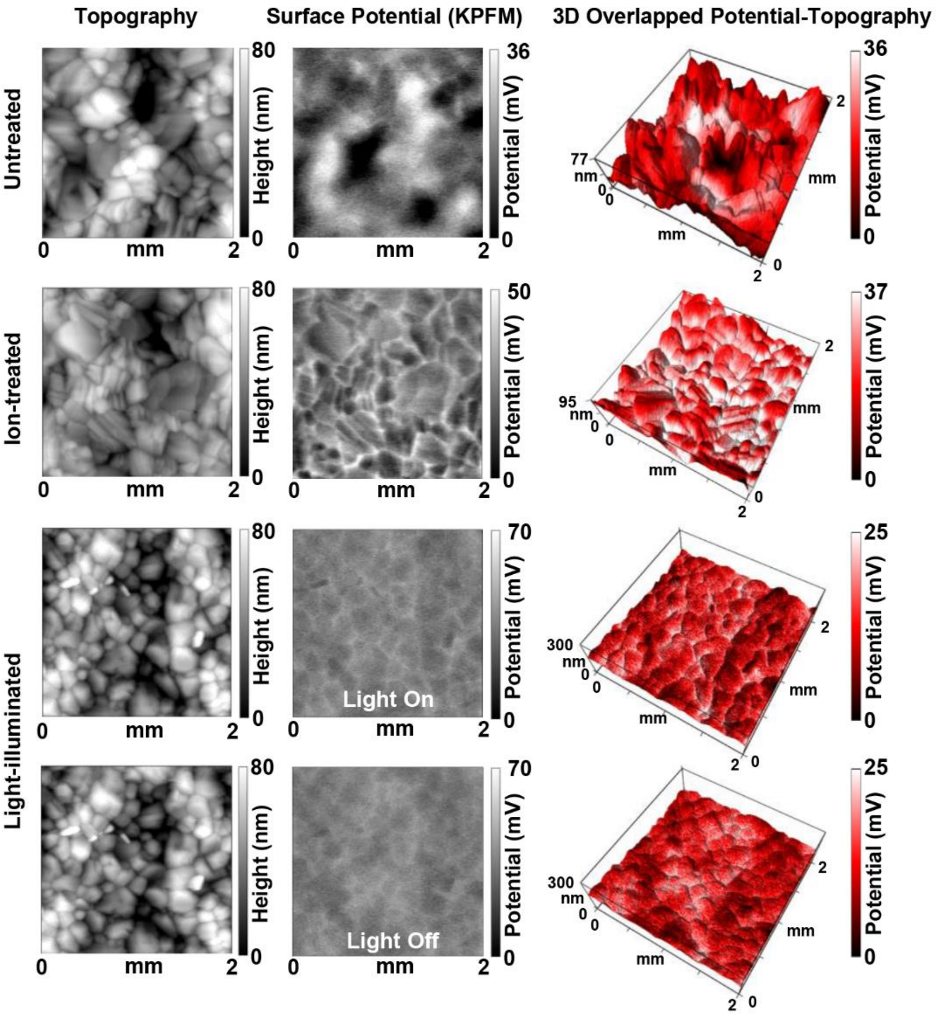

Supplementary Figure 13. Topographies (first column) and surface charge density profiles (second column) of untreated (first row), Ion-treated (second row), and light-illuminated (third and fourth row) $MA_{0.6}FA_{0.4}PbI_{2.9}Br_{0.1}$ perovskite film. The images in the third and fourth row were obtained from light on and off during the measurement, respectively. The images in the third column show 3D plots of topographies colored based on the surface potential values. Both images of light illuminated cases show clear charge trapping along grain boundaries, but the charge trap is more contrasted when KPFM operation is under the light on.

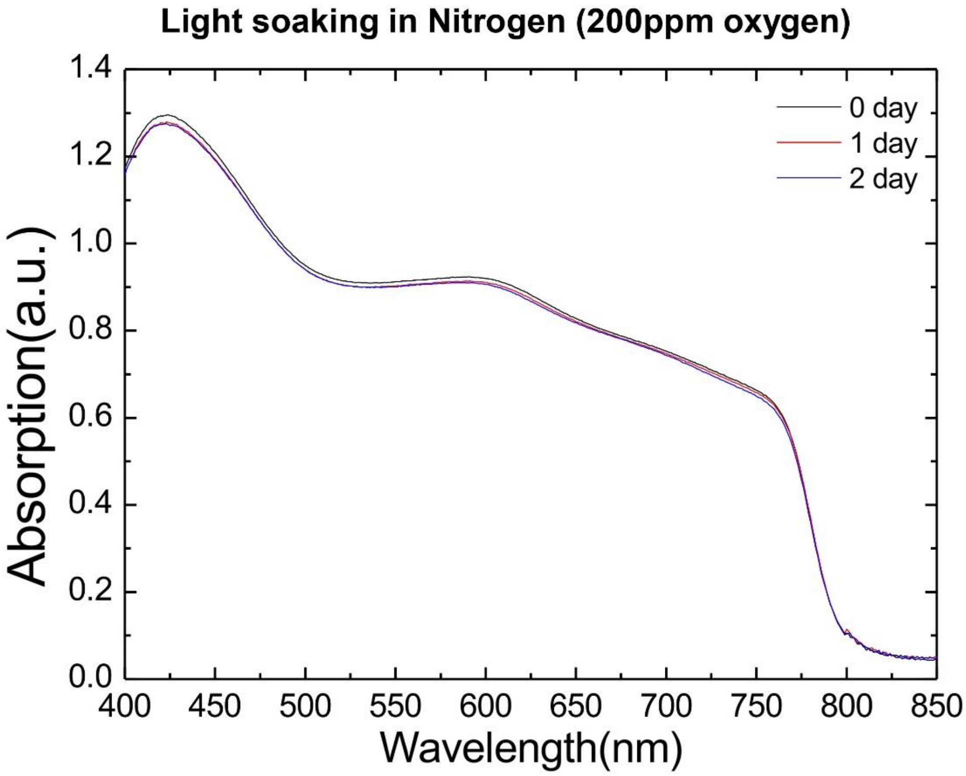

Supplementary Figure 14. Absorption spectra of the perovskite film under one sun illumination in nitrogen. Light was illuminated in a moisture-free condition.

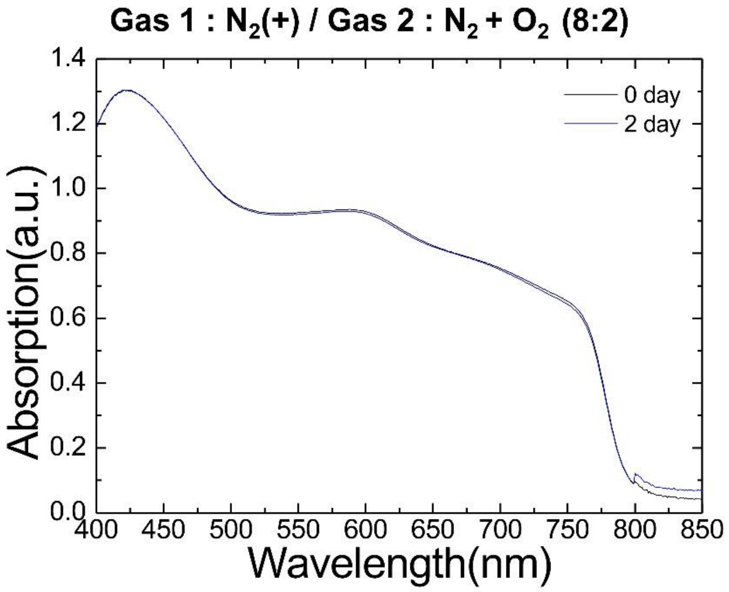

Supplementary Figure 15. Absorption spectra of the perovskite film in dry air after deposition of positive nitrogen ions.

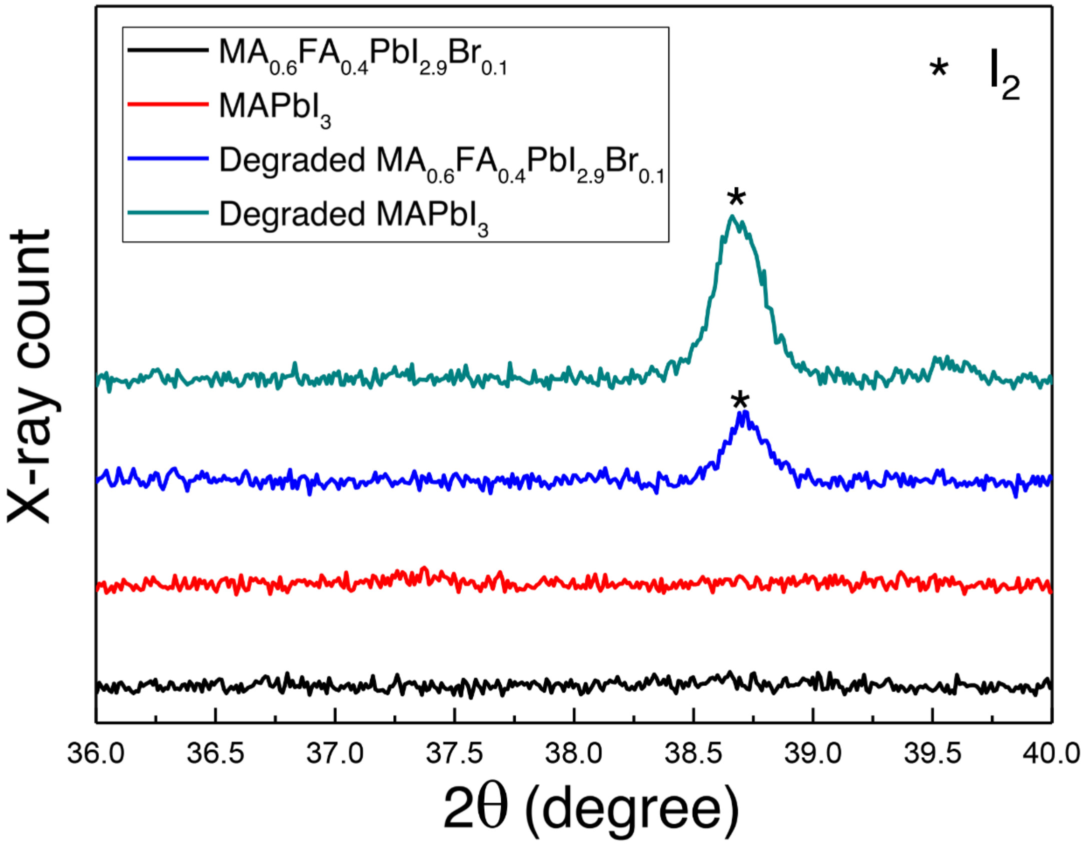

Supplementary Figure 16. X-ray diffraction (XRD) patterns of the fresh and degraded samples of $MAPbI_3$ and $MA_{0.6}FA_{0.4}PbI_{2.9}Br_{0.1}$. The graph shows the magnified XRD patterns around the peak originating from I_2 (*).

		Flow rate (L m^{-1})	Current of generated ion (pA)	Applied voltage
Gas1	Nitrogen	2	20.6 ~ 25.8	4.2kV(0.009 mA)
	Hydrogen	2	-3.2 ~ -4.8	-1.55kV(-0.4 mA)
Gas2	Nitrogen	1.5		
	Dry air	1.5	-	

Supplementary Table 1. The parameters for ion generation and data obtained from MFC controller and Faradaycup.

Supplementary Note 1 : Optimization of mixed $MA_xFA_{1-x}PbI_yBr_{3-y}$

In a mixed cation and halide anion system of Pb-based perovskite, $APbX_3$, the Goldschmidt tolerance factor t is defined in terms of the average radii of ions as follows.

$$t = \frac{\overline{r_A}+\overline{r_X}}{\sqrt{2}(\overline{r_{Pb}}+\overline{r_X})} \quad \text{(Supplementary Equations 1)}$$

where $\overline{r_A}$, $\overline{r_X}$, and $\overline{r_{Pb}}$ are the average radii of the cation, anion, and lead ions, respectively. The I^-, Br^- and Pb^{2+} ions have a spherical shape and the radii of these ions have been measured in previous works[1]. In constrast, it is difficult to precisely estimate the radii of the organic cations perched on the A site due to their non-spherical shape and rotational motion, nevertheless, several groups already reported the calculated effective ion radius of both MA+ and FA+ cation[2,3,4,5]. By using equation 1 and the suggested ionic radii (r_{MA+}= 0.18 nm, r_{I-}= 0.22 nm, r_{Pb2+}= 0.12nm), the tolerance factor of $MAPbI_3$ is calculated to be 0.83[5], indicating that MA+ cations are too small to fit into the interstices between PbX_6 octahedra. This mismatch causes crystal distortion, and consequentially $MAPbI_3$ perovskite have unstable tetragonal crystal structure[6,7]. By partially replacing MA+ and I- to relatively larger FA+ and smaller Br- ions respectively, crystal distortion can be alleviated to produce more stable cubic structure with the tolerance factor between 0.9-1[8]. Simple calculation reveals that replacing I- to Br-(r_{Br-}= 0.196 nm) only marginally affects the perovskite crystal structure as the tolerance factor of $MAPbBr_3$ is only 0.01 higher than that of $MAPbI_3$. On the other hand, replacing MA to FA cation can significantly alter the crystal structure because FA cations are expected to be much larger than MA cations. The exact radius of FA cation is still controversial[8], but considering that $FAPbI_3$ can possess not only non-perovskite

yellow δ-phase with hexagonal crystal structure (t >1) but also black perovskite α-phase with cubic structure (0.9 < t < 1), it can be speculated that the tolerance factor of $FAPbI_3$ would be around 1. The radius of FA cation r_{FA+} is estimated to be around 0.26 nm from these speculations and equation 1. The tolerance factor of mixed cation system, $MA_xFA_{1-x}PbI_3$, is calculated as a function of the ratio x as shown in Supplementary Fig. 1. This relation suggests that the x values between 0.2 and 0.6 lead to the most stable cubic crystal structure, as the resulting tolerence factor lies between 0.9 and 1.

We measured X-ray diffraction(XRD) patterns of five types of mixed perovskite films (*x*= 1, 0.8, 0.6, 0.4, 0.2) coated on the ITO glass in order to compare their stability. Supplementary Fig. 2a,b show the XRD patterns of the fresh and degradaded samples. The perovskite films were degraded in the chamber (relative humidity (RH) ~ 50%) for 10 hours under one sun illumination. The peak originating from PbI_2 at 12.7 degrees intensively appears in the case of $MAPbI_3$ (*x*=1), and the peak of non-perovskite δ-phase $FAPbI_3$ at 11.7 degrees appears for x < 0.4, which indicates severe instability to water vapor[10,11]. (Supplementary Fig. 2c,d) These observations suggest that the most stable composition would be $MA_{0.6}FA_{0.4}PbI_3$ (*x*=0.6), which still posesses the tolerance factor ensuring the cubic crystal structure.

We also fabricated the full devices with the ITO/C_{60}/Perovskite/Spiro-MeOTAD/Au structure in order to find the optimal composition with respect to the photovoltaic performances in mixed perovskite $MA_xFA_{1-x}PbI_yBr_{3-y}$. Supplementary Fig. 3a,b summarize the PCEs as a function of the MA+ fraction(x) and the Br- fraction(3-y), respectively. As a result, the $MA_{0.6}FA_{0.4}PbI_{2.9}Br_{0.1}$–based devices were shown to the best performance. Considering that MA:FA=0.6:0.4 is the best composition for stability,

we concluded that $MA_{0.6}FA_{0.4}PbI_{2.9}Br_{0.1}$ would be the best composition in terms of both performance and stability in mixed perovskite systems.

.

Supplementary Note 2: Experimental setup for ion generation and deposition[11]

The whole chamber contains two connected chambers: the ion generation(IG) chamber and the ion deposition(ID) chamber. The IG chamber has cylindrical shape with 30 mm diameter and and 35 mm height. It is made of transparent acrylic which makes it possible to see the state of corona discharge during experiment. Stainless steel pin and plate creates highly asymmetric electric field in the chamber, when a bias voltage is applied between the pin and the plate. Gas 1 can flow into the chamber through an inlet on the side wall. The polarity of generated ions is determined by the polarity of applied voltage to the pin. The current of generated ions was measured by Faradaycup electrometer (Keithley Sub-femtoamp romote sourcemeter, 6430). A high-voltage supply (FuG ElektronikGmbH, HCP140-12500) apply voltage to the pin and the substrate in the ID chamber.

The ions generated in the IG chamber flows through a 115 mm long pipe with a 1.5mm diameter that connects the IG and ID chambers. A tee tube connected to this pipe introduces Gas 2 into the system. The flow rates of Gas 1 and 2 are both controlled by mass flow controllers (MKS instruments, MFC Controller 247D, MFC 1179A). The negatively (postively) charged gas ions are electrostatically attracted and deposited on the positively (negatively) biased substrate with the bias voltage of 2kV (-2kV).

Corona ion generation

Nitrogen gas inflow with the flow rate of 2 lpm was transformed into positive nitrogen ions by applying 4.2 kV to the pin. The electric current of generated nitrogen ions was measured to be 20.6-25.8 pA, which is indicative of positive ion generation. To generate negative ions, we used hydrogen gas with the flow rate of 2 lpm and applied negative bias (-1.55 kV) to the pin, which generates the current of -3.2 ~ -4.8 pA. (see **Supplementary Table 1**)

Atmosphere control

To maintain the condition of the air inside the chamber at constant, the flow rate of Gas 2 was controlled by MFC and set as 1.5 lpm throughout the measurements. Nitrogen, dry air, humidified nitrogen and air were used as Gas 2. Nitrogen and hydrogen gases are highly purified by 99.999%, and dry air consists of 80% of nitrogen (99.999%) and 20% of oxygen (99.995%). Gas 2 passes through a water bubbler that controls the humidity in the chamber. The relative humidity was measured by portable multifunction data-logger (Delta OHM, Data logger DO9847, Temp&Humidity probe HP474AC) at the gas exit of deposition chamber.

Supplementary Note 3: Topography and Kelvin probe force microscopy

All the samples for topography and Kelvin probe force microscopy measurements were prepared on ITO glass substrates. The perovskite films were spin-coated on the ITO glass and ITO/C_{60} substrate. The ion-treated sample was prepared by depositing N_2 positive corona ion for 1 hr on the ITO/perovskite substrate. In the case of the light-illuminated sample, the ITO/C_{60} substrate was used in order to measure positive charge accumulation profile. After one sun illumination for 1 hr, the sample was measured under light on or off during KPFM operation (See Supplementary Fig. 9).

Topography and Kelvin probe force microscopy (KPFM) signals were measured by using an atomic force microscope (MFP-3D, Asylum Research, USA) with a Pt-coated tip with the spring constant of 2 nN nm^{-1} and the resonant frequency of 77 kHz. For each line scanning, topography was first measured and successively the surface potential was measured while scanning the same line at a fixed distance above the sample surface. The tip was positioned sufficiently away from the substrate (as far as 20 nm) in order to exclude the corss-talk artefacts from topography footprints[12]. The surface potential was measured using an active electronic feedback circuitry: the bias voltage to the tip was modulated in order to equate the potential of the tip with that of the surface, resulting in minimum vibration amplitude of the AFM tip at the fundamental frequency. A 150W halogen bulb with single fiber light guide was used for illumination.

Supplementary references

1 Shannon, R. Revised effective ionic radii and systematic studies of interatomic distances in halides and chalcogenides. *Acta Crystallographica Section A* **32**, 751-767 (1976).

2 Amat, A. *et al.* Cation-induced band-gap tuning in organohalide perovskites: interplay of spin–orbit coupling and octahedra tilting. *Nano letters* **14**, 3608-3616 (2014).

3 Cohen, B. N., Labarca, C., Davidson, N. & Lester, H. A. Mutations in M2 alter the selectivity of the mouse nicotinic acetylcholine receptor for organic and alkali metal cations. *The Journal of general physiology* **100**, 373-400 (1992).

4 Dimesso, L., Quintilla, A., Kim, Y. M., Lemmer, U. & Jaegermann, W. Investigation of formamidinium and guanidinium lead tri-iodide powders as precursors for solar cells. *Materials Science and Engineering: B* **204**, 27-33 (2016).

5 Green, M. A., Ho-Baillie, A. & Snaith, H. J. The emergence of perovskite solar cells. *Nature Photonics* **8**, 506-514 (2014).

6 Wang, Y. *et al.* Density functional theory analysis of structural and electronic properties of orthorhombic perovskite CH3NH3PbI3. *Physical Chemistry Chemical Physics* **16**, 1424-1429, (2014).

7 Kumar, G. R. *et al.* Phase transition kinetics and surface binding states of methylammonium lead iodide perovskite. *Physical Chemistry Chemical Physics* **18**, 7284-7292, (2016).

8 Li, Z. *et al.* Stabilizing Perovskite Structures by Tuning Tolerance Factor: Formation of Formamidinium and Cesium Lead Iodide Solid-State Alloys. *Chemistry of Materials* **28**, 284-292, (2016).

9 Stoumpos, C. C., Malliakas, C. D. & Kanatzidis, M. G. Semiconducting Tin and Lead Iodide Perovskites with Organic Cations: Phase Transitions, High Mobilities, and Near-Infrared Photoluminescent Properties. *Inorganic Chemistry* **52**, 9019-9038, (2013).

10 Lee, J.-W. *et al.* Formamidinium and Cesium Hybridization for Photo- and Moisture-Stable Perovskite Solar Cell. *Advanced Energy Materials* **5**, 1501310, (2015).

11 Chang, J. S., Lawless, P. A. & Yamamoto, T. Corona discharge processes. *IEEE Transactions on Plasma Science* **19**, 1152-1166, (1991)

12 Barbet, S. *et al*. Cross-talk artefacts in Kelvin probe force microscopy imaging: A comprehensive study. *Journal of Applied Physics* **115**, 144313, (2014).
